# Exploratory assessment of parental physical disease categories as predictors of documented physical child abuse

**DOI:** 10.1007/s00431-023-05317-1

**Published:** 2023-11-13

**Authors:** Troels Græsholt-Knudsen, Charlotte Ulrikka Rask, Steven Lucas, Bodil Hammer Bech

**Affiliations:** 1 Research Unit for Mental Public Health, Department of Public Health, Aarhus University, Bartholins Allé 2, 8000 Aarhus C, Denmark; 2 Department of Forensic Medicine, Aarhus University, Palle Juul-Jensens Boulevard 99, 8200 Aarhus N, Denmark; 3 Department of Child and Adolescent Psychiatry, Aarhus University Hospital Psychiatry, Palle Juul Jensens Boulevard 175, 8200 Aarhus N, Denmark; 4 Department of Clinical Medicine, Aarhus University, Olof Palmes Allé 43, 8200 Aarhus N, Denmark; 5 Department of Women’s and Children’s Health, Uppsala University, Akademiska Sjukhuset, 751 85 Uppsala, Sweden; 6 Research Unit for Epidemiology, Department of Public Health, Aarhus University, Bartholins Allé 2, 8000 Aarhus C, Denmark

**Keywords:** Child, Maltreatment, Physical child abuse, Parental health, Risk factor, Prediction

## Abstract

Improved prediction of physical child abuse could aid in developing preventive measures. Parental physical disease has been tested previously as a predictor of documented physical child abuse but in broad categories and with differing results. No prior studies have tested clinically recognizable categories of parental disease in a high-powered dataset. Using Danish registries, data on children and their parents from the years 1997–2018 were used to explore several parental physical disease categories’ associations with documented physical child abuse. For each disease category, survival analysis using pseudovalues was applied. When a parent of a child was diagnosed or received medication that qualified for a category, this family and five comparison families not in this disease category were included, creating separate cohorts for each category of disease. Multiple analyses used samples drawn from 2,705,770 children. Estimates were produced for 32 categories of physical diseases. Using Bonferroni-corrected confidence intervals (CIc), ischemic heart disease showed a relative risk (RR) of 1.44 (CIc 1.13–1.84); peripheral artery occlusive disease, RR 1.39 (CIc 1.01–1.90); stroke, RR 1.19 (1.01–1.41); chronic pulmonary disease, RR 1.33 (CIc 1.18–1.51); ulcer/chronic gastritis, RR 1.27 (CIc 1.08–1.49); painful condition, 1.17 (CIc 1.00–1.37); epilepsy, RR 1.24 (CIc 1.00–1.52); and unspecific somatic symptoms, RR 1.37 (CIc 1.21–1.55). Unspecific somatic symptoms were present in 71.87% of families at some point during the study period.

*Conclusion*: Most parental physical disease categories did not show statistically significant associations, but some showed predictive ability. Further research is needed to explore preventive potential.

**Table:** 

**What is Known:** *• Few and broad categories of parental physical disease have been examined as risk factors for severe physical child abuse; no prior study has used several categories as predictors.*	
**What is New:** *• Unspecific symptoms, ischemic heart disease, peripheral artery occlusive disease, stroke, chronic pulmonary disease, stomach ulcer/chronic gastritis, painful condition, and epilepsy all showed to be potential predictors, with unspecific symptoms being the most prevalent.*

**Supplementary Information:**

The online version contains supplementary material available at 10.1007/s00431-023-05317-1.

## Introduction

Child physical abuse has detrimental consequences across the life span and is recognized by the World Health Organization as an important target for preventive efforts [1]. A prevailing etiologic explanation of physical child abuse is family stressors overcoming supportive factors [2]. In family stress theory [3], family stress has been connected with chronic illness, both among adults and children [4]. Adult disease has been linked with perceived stress [5], and parental chronic illness has been associated with deficiencies in family cohesion and functioning and increased levels of conflict [6]. Additionally, a dose–response relationship between symptom severity and negative impact on family functioning has been indicated [6]. Parental mental health problems have been found to predict lethal child abuse [7, 8], but parental physical health has shown differing results [9–11]. In the only study investigating parental physical health severity, using the parental Charlson Comorbidity Index and published concurrently in this journal, our group found no association with documented physical child abuse [12]. Possibly, this result was due to few high index scores among parents, with only 1.7% scoring ≥ 2, and because heterogeneous categories of diseases were covered. Extending beyond the severe categories in the Charlson index, adult disease has been categorized, based on previous studies [5]. Despite high prevalence [13, 14], diagnoses not attributable to well-defined physical diseases were not included [5]. These diagnoses, comprising functional somatic syndromes [15], known to be associated with stress [16] and adverse life effects [17, 18], and diagnoses defined by their symptoms and not pathology, together represent unspecific somatic symptoms.

In this study, we use categories for physical disease, with the addition of unspecific somatic symptoms [19, 20], to explore the association between parental physical health problems and documented physical child abuse. As parental physical health severity did not show a causal association with the outcome, the current study is exploratory.

## Methods

A prospective, observational cohort study was carried out. The data source used is also used in our article [12], published concurrently in this journal. All children living in Denmark between the 1st of January 1997 and the 31st of December 2018 and their legally registered parents were included, choosing this study period because among others the Fertility Database and the Medical Birth Register were available from this date. A parent was defined here as an adult registered as the current legal parent of a child, including unrelated adults in a legal parenting role. Any children without legally registered parents were excluded. Any children who emigrated and re-entered Denmark were censored at time of emigration to avoid immortality bias. Background data were drawn from other registries (see Table 1).

**Table 1:** List of variables, their study definitions, sources, and levels

Variable	Registry source	Study definition
Child age	Fertility Database [21], Population Register, Medical Birth Register [22]	Child age at inclusion, as recorded in population registers
Calendar year group		Calendar year group at inclusion
Mean parental age	Fertility Database, Population Register, Medical Birth Register	Parental or guardian age at inclusion; in case of more than one parent or guardian, the mean of ages was computed. In model 2, as legal guardians could change during study participation, this is treated as a time-varying covariate
Neighborhood resources	Income Register [23], Parish Register, Population Register, Address Register, Addresses in Denmark Register (except for Parish Register, the remaining registers are all connected to the population register)	Neighborhood homogeneity as measured from family total income, inspired by Cheung et al. [24]. The variable was in thousands, the difference between the highest and the lowest quartile of average family income through the last 3 years, among families with children in the parish, or clustering of parishes according to another study [25], and subtracting the yearly rate for relative poverty [26, 27]
Number of children in family^a^	Population Register, Central Personal Register Status	The number of other children with the same parent(s) as the child, inspired by multiple studies [28–30]
Maltreatment of parent as a child^b^	Children and Young Adults (preventive actions) Register, Danish National Patient Register and related^c^, Victims of Criminal Offences Register [31]	Welfare services interventions, child health records, and criminal records joined in a variable indicating presence of either maltreatment or neglect, inclusion inspired by Widom et al. [32]
Immigration background (ethnicity)	Fertility Database, Population Register [33], Residence Permit Register [34]	Country of birth or citizenship for parents and children, immigrant was defined as at least one parent originating from a foreign country, inclusion was inspired by multiple studies [29, 35–37]
Status as refugee	Residence Permit Register	The familial basis of residence in Denmark, as available in the registries; any family granted permanent or temporary residency as refugees, based on the right to asylum or similar, or residency based on humanitarian grounds or risk assessment, were classified as refugees. This variable was included based on expert opinion (H.N. Christensen, personal communications, 2019)
Reconstituted family	Fertility Database, Population Register	Whether child was living with biological parents only, was adopted, or living with a stepparent—this variable, along with the connection between child and parents, was updated each year instead of each month, inclusion was inspired by Schnitzer et al. [38]
Education^d^	Education Register [39]	Highest level of finished education among the parents, the ISCED classification was used to make a classification of the highest educated adult in the household, inclusion inspired by multiple studies [28, 29]
Income	Income Register	Mean family income during last 3 years as a continuous variable measured in hundreds, subtracting the yearly rate for relative poverty [26] so that a negative income meant an income less than the current poverty level in that year, inclusion inspired by Paxson et al. [40]
Parental psychiatric disease^d^	Danish National Patient Register and related^c^	Diagnosis of psychiatric disorders or dispensation of medication for psychiatric disorders, drawn from the patient register and the register of drug prescriptions. This was a cluster of variables, as described by Prior [5], and modified, adding personality disorder as described in the algorithm for exposure. Psychological distress was not used because of a broad definition. The categories for alcohol problems and other substance abuse were described separately. Inclusion was inspired by multiple studies [7, 8]
Inter-parental violence	Danish National Patient Register and related^c^, Victims of Criminal Offences Register	Observations of violence by the police in conjunction with health data on injuries. This was a dichotomous variable indicating whether or not inter-parental violence has ever taken place and could go from negative to positive, but not back to negative, as inter-parental violence is known to have effects on risk lasting several years [41]. For codes used, see our code [42]. Inclusion inspired by Jobe-Shields et al. [43]
Parental drug abuse^e^	Danish National Patient Register and related^c^	Any drug-related diagnosis and any crime related to drug possession or intake—was a dichotomous variable indicating abuse or no abuse. The literature does not carry precise instructions about whether former substance abuse carries increased risk at present [7, 44–46]. We chose a partly arbitrary limit of 2 years [5] to allow for presumed differing risks among current and former substance abusers and the fact that ongoing substance abuse may occur with no indications in the registries. Classification was inspired by Ahacic et al. [47] and inclusion by Wasserman et al. [46]
Parental alcohol abuse^e^	Danish National Patient Register and related^c^	Any alcohol-related diagnosis and any crime related to alcohol intake—was a dichotomous variable indicating abuse or no abuse and going 2 years back, same arguments as for drug abuse, inclusion inspired by Gessner et al. [29]

The studies cited provided findings to justify the inclusion of the variables. Table 1 is identical to the table included in our article [12], published concurrently in this journal

^a^This variable was based on number of children with the same parents (including single parents) instead of the planned definition due to unexplained inaccuracies in the dataset. When using the original sources, in some instances, data indicated cohabitation of several hundred adults and children

^b^Registrations of maltreatment in ICD 8 is less detailed than ICD 10—see our preregistration [42]. This variable was collapsed to avoid small cells in the analysis

^c^Danish National Patient Register(in- and outpatient contacts) [48], causes of death register[49]

^d^This variable was collapsed to avoid small cells in the analysis

^e^These were collapsed into parental substance abuse to avoid small cells in the analysis

### Exposure

See Table 2 for an overview. Parental disease was categorized mainly according to Prior et al. [5]. To ensure optimal coverage in the dataset, diagnoses not categorized by Prior et al. were added to existing categories (see below). The sorting algorithm was inspired by other studies [19, 20]. The full algorithm, and the code for data analysis, is described in the pre-registration for this study [42].

**Table 2:** Disease clusters and categories, modified from Prior et al. [5]

Cluster	Disease category	Diagnosis time frame^a^	ATC drug codes used^b^
Circulatory system	Hypertension	Ever	
	Dyslipidemia	Last 2 years	C10AA, C10BA, C10AX, C10AB, C10AC, C10AD
	Ischemic heart disease	Ever	C01DA
	Atrial fibrillation	Ever	
	Heart failure	Ever	
	Peripheral artery occlusive disease	Ever	
	Stroke	Ever	
Endocrine system	Diabetes mellitus	Ever	A10A, A10B
	Thyroid disorder	Last 2 years	H03
	Gout	Ever	
Pulmonary system and allergy	Chronic pulmonary disease	Ever	
	Allergy	Last 2 years	
Gastrointestinal system	Ulcer/chronic gastritis	Ever	
	Chronic liver disease	Ever	
	Inflammatory bowel disease	Ever	
	Diverticular disease of intestine	Ever	
Urogenital system	Chronic kidney disease	Ever	
	Prostate disorders	Ever	
Musculoskeletal system	Connective tissue disorders	Ever	
	Osteoporosis	Ever	M05B, H05AA, G03XC01
	Painful condition	Last year	N02BA51, N02BE, M01A, M02A
Hematological system	Anemias	Last 2 years	
Cancers	Cancer	Last 5 years	
Neurological system	Vision problem	Ever	
	Hearing problem	Ever	
	Migraine	Last 2 years	N02C
	Epilepsy	Ever	
	Parkinson’s disease	Ever	
	Multiple sclerosis	Ever	
	Neuropathies	Last 2 years	
Unspecific symptoms	Unspecific symptoms	Last 2 years	
	Unspecific symptoms	Ever	
Other diagnoses^c^	Any disease not classifiable under the categories mentioned above and not classified as trauma, infections, and all birth-related conditions	Last 3 years	

^a^Diagnosis time frame is during study period and 3-year burn-in. For example, if a parent at any time during the study has a code for anemia within the last 2 years, this family is exposed for this condition. If a parent at any time during the study receives a diagnosis for hypertension, this family remains exposed in this category for the remainder of the study

^b^All categorizations by drug prescriptions needed at least two prescriptions within the last year, except for the disease category “Painful condition” which needed at least four prescriptions within the last year

^c^All other disease classifications not included in any other category, and not excluded according to the algorithm as described in the pre-registration (does not contain trauma, birth-related conditions and infections)

In short, all ICD 10 diagnoses were extracted from the National Patient Register; accidents, infections, and procedures were left out, and all remaining diagnoses, except psychiatric diagnoses (F-diagnoses), were sorted into conventionally defined diagnoses and unspecific symptoms. The conventionally defined diagnoses were then filtered according to Prior et al. [5] but excluding the category HIV/AIDS as our focus did not include infections. Finally, a category of “Other diagnoses” was added, comprising all diagnoses that did not fit within the well-defined disease categories or the category of unspecific symptoms, were not F-diagnoses, and were not excluded as described above. This resulted in 384 diagnosis sorting rules in addition to Prior et al., made by the first author, who is a trained medical doctor. To ease the reproducibility of this study, the final classification of 11,866 diagnostic codes is available in the Online Resource 1. For some categories, additional information on redeemed prescriptions was used, inspired by Prior et al. [5]. This captured a number of diseases treated in the primary sector. Information on redeemed prescriptions was obtained from the Register of Medicinal Products Statistics. For all disease categories, a 3-year burn-in period before the study was established. Health information for parents were read in from the first of January 1994 and onward, and, for example, parents with a code for hypertension during the period 1994–1997 were already classified in this category at study entry. All categories were updated throughout the study period on a monthly basis. To ensure precise categorization, some prescriptions used in the original categorization by Prior et al. were left out. For example, Anatomical Therapeutic Chemical (ATC) code C02, which was used by Prior et al. to categorize hypertension, is also registered for use in attention-deficit hyperactivity disorder and prostate hyperplasia. Prior et al. included this code because of its most prevalent use (A. Prior, personal communications, 2020), but we have excluded it for greater specificity.

### Outcome

The outcome was documented physical child abuse. All hospital and police codes that indicated violence against a child, including lethal violence, were combined into a dichotomous variable indicating the first incidence of abuse. Details are available in our description of the coding algorithm [42].

### Covariates

Covariates were chosen based on the literature (see Table 1 for references and data sources). Parental psychiatric diseases were extended from Prior’s categories to also include personality disorder [7, 50].

### Statistical methods

We used pseudoobservations [51] to estimate relative risks (RR) adjusted for covariates, with 95% confidence intervals (CI) and Bonferroni-corrected 95% confidence intervals (CIc) [52, 53]. A child with at least one parent in a specific disease category was identified as exposed and the index date set at parental time of diagnosis or birth date of the child, whichever came last. Then, for each child exposed, five children who were not exposed at the index date were drawn from the underlying source population, matching on date of inclusion (a child is identified as exposed and within 3 months of calendar time, five other children not exposed are drawn), reconstituted family (living with biological parents, living with one or more unrelated adults, adopted or in foster care), child ages/birth year (matched within 1 year), number of children in the family (either one child, two children, three to five children, or six or more children), and mean parental age (within 5 years). Thus, individual cohorts were created for all disease categories studied. Each family was followed until end of study period, first incident of documented physical child abuse, emigration, the child being without registered parents, dying or reaching their 18th birthday, whichever came first, allowing for at least 1 month and at most < 18 years between exposure and outcome. Children experiencing the outcome before the exposure were excluded. Each child could participate in more than one cohort, but not twice in the same cohort, and not after first incident of documented physical child abuse. A full model, containing pre-specified variables, and a parsimonious model, reduced to avoid collinearity and small cells, were produced for all analyses. To avoid computational overload, a maximum of 100,000 exposed children were allowed in each analysis, drawn randomly among those available. Only rows with information on all variables were included. The parental disease categories were not adjusted among each other. Pseudoobservations assume that censoring in the dataset is marginally independent. As this was not the case for the calendar time groups, the dataset was stratified on this variable and pseudovalues generated within these strata. The same child could be included both as exposed and un-exposed; Eicker-Huber-White standard errors were used to compensate for this. Siblings could be included, and therefore clustering was adjusted for on a family level. In all models, death not related to abuse was treated as a competing risk. Analyses were presented with and without Bonferroni corrections, correcting for tests of 33 categories.

## Results

### Samples, demographics, and events

Exposed and unexposed children were drawn from 2,705,770 children available in the dataset. First-time documented physical child abuse was experienced by 70,892 children during the study period, and lethal child abuse was experienced by 111 children. Table 3 describes the full population’s distribution across covariates; this table is derived from the table included in our article [12], published concurrently in this journal. Table 4 describes the risk time, events, number of children studied, and number and percentage of children exposed in the full cohort. Most disease categories had less than 10% exposed during the study period, except unspecific symptoms (72%), allergy (23%), painful condition (11%), and other diagnoses (69%) (Table 4).

**Table 3:** Source population characteristics at entry in the source population

Characteristics	Total population *N*: 2,705,770
Child age, y, median (quartile 1; quartile 3)^a^	0.0 (0.0; 7.1)
Calendar year group, *n* (%)	
1997–2002	1,605,574 (59%)
2003–2009	508,413 (19%)
2010–2018	591,783 (22%)
Mean parental age, y, mean (SD)	34.38 (6.64)
Missing entries	17,260 (0.6%)
Neighborhood resources, thousand Euros, mean (SD)	106.76 (37.8)
Missing entries	552,251 (20%)
Number of children in family, *n* (%)	
One child	1,247,119 (46%)
Two children	1,004,042 (37%)
Three to five children	443,142 (16%)
Six or more children	11,467 (0.4%)
Parental maltreatment in childhood, *n* (%)	
No maltreatment or neglect	2,536,028 (94%)
Maltreatment or neglect, one or both parents	169,742 (6.3%)
Immigration background (ethnicity), *n* (%)	
No foreign parents	1,794,749 (66%)
One or more foreign parents	589,569 (22%)
Missing entries	321,452 (12%)
Status as refugee, *n* (%)	
Not in need of protection	2,624,427 (97%)
In need of protection	81,343 (3.0%)
Reconstituted family, *n* (%)	
Living with biological parent(s)	1,968,517 (73%)
Living with one or more unrelated adults	73,920 (2.7%)
Adopted or in foster care	40,115 (1.5%)
Missing entries	623,218 (23%)
Family highest education, *n* (%)	
Primary or secondary education	1,483,929 (55%)
Tertiary education or higher	930,910 (34%)
Missing entries	290,931 (11%)
Family income, thousand Euros, mean (SD)	123.13 (243.99)
Missing entries	363,695 (13%)
Parental psychiatric disease, *n* (%)	
No psychiatric disease	2,609,755 (97%)
Any psychiatric disease except substance abuse	96,015 (3.6%)
Inter-parental violence, *n* (%)	
No inter-parental violence	2,705,052 (99%)
Inter-parental violence	718 (0.0%)
Parental substance abuse, *n* (%)	
No parental substance abuse	2,683,304 (99%)
Any parental substance abuse	22,466 (0.8%)

^a^Rounded to first decimal

**Table 4:** Follow-up time, events, and number of children in each model

Cluster	Disease category	Mean follow-up time, years	Total follow-up time, years	Abuse cases	Lethal abuse cases^a^	Non-abuse deaths^a^	Children analyzed in the model^b^	Exposed in source population, absolute and (percentage)
Circulatory system	Hypertension	12.76	7,445,895	13,085	9	468	583,690	148,107 (5.5%)
	Dyslipidemia	13.59	7,705,366	11,481	7	369	567,164	202,248 (7.5%)
	Ischemic heart disease	12.39	4,279,693	8590	< 6	284	345,487	70,422 (2.6%)
	Atrial fibrillation	12.74	2,309,776	3816	< 6	141	181,349	33,511 (1.2%)
	Heart failure	12.93	1,019,768	1782	< 6	63	78,866	15,519 (0.6%)
	Peripheral artery occlusive disease	12.46	2,026,085	3783	< 6	133	162,615	30,376 (1.1%)
	Stroke	12.57	3,382,855	6615	< 6	226	269,091	50,266 (1.9%)
Endocrine system	Diabetes mellitus	12.09	7,065,807	13,148	17	461	584,290	135,194 (5.0%)
	Thyroid disorder	12.06	6,982,573	11,793	21	459	578,802	181,242 (6.7%)
	Gout	12.50	960,135	1934	< 6	72	76,817	14,164 (0.5%)
Pulmonary system and allergy	Chronic pulmonary disease	11.89	7,000,189	13,986	17	564	588,885	173,546 (6.4%)
	Allergy	12.15	6,989,338	13,803	22	584	575,442	633,093 (23%)
Gastro-intestinal system	Ulcer/chronic gastritis	11.97	3,188,274	7725	9	256	266,431	56,900 (2.1%)
	Chronic liver disease	11.97	1,090,709	2660	< 6	92	91,101	17,538 (0.7%)
	Inflammatory bowel disease	11.72	4,202,999	7557	< 6	340	358,575	67,226 (2.5%)
	Diverticular disease of intestine	13.42	2,130,161	2842	< 6	81	158,754	28,730 (1.1%)
Urogenital system	Chronic kidney disease	12.34	967,055	1568	< 6	68	78,338	15,507 (0.6%)
	Prostate disorders	12.20	850,802	1605	< 6	42	69,763	13,624 (0.5%)
Musculo-skeletal system	Connective tissue disorders	12.05	5,028,212	8907	14	367	417,310	79,537 (2.9%)
	Osteoporosis	13.46	2,533,409	3136	< 6	97	188,203	35,685 (1.3%)
	Painful condition	12.79	7,321,375	14,784	12	470	572,498	303,268 (11%)
Hematological system	Anemias	12.73	3,141,587	5312	6	199	246,793	54,925 (2.0%)
Cancers	Cancer	12.39	7,197,549	12,551	10	491	580,891	133,656 (4.9%)
Neurological system	Vision problem	12.34	7,235,867	11,558	6	488	586,512	147,407 (5.5%)
	Hearing problem	12.86	7,525,399	11,277	10	411	585,241	159,521 (5.9%)
	Migraine	12.49	7,182,776	13,516	14	481	575,039	188,450 (7.0%)
	Epilepsy	11.71	2,755,407	6,412	14	244	235,209	44,647 (1.7%)
	Parkinson’s disease	12.59	118,508	186	< 6	< 6	9412	1786 (0.1%)
	Multiple sclerosis	11.86	1,212,037	2358	6	111	102,191	18,413 (0.7%)
	Neuropathies	12.69	7,323,510	13,792	18	467	577,151	178,240 (6.6%)
Unspecific symptoms	Unspecific symptoms, two years back	11.72	6,780,175	13,344	20	619	578,457	1,799,771 (67%)
	Unspecific symptoms, ever	11.49	6,674,713	12,021	15	639	581,146	1,944,689 (72%)
Other diagnoses	Other	11.47	6,650,071	13,664	8	586	580,009	1,858,098 (69%)

^a^Counts less than 6 were censored due to privacy regulations from Statistics Denmark

^b^Categories approaching 600,000 does so because this represents the maximum of children allowed in the model (100,000 exposed and 500,000 controls), subtracted for those cases that could not be compared with 5 children

### Estimates of relative risk

Table 5 describes the results for each disease category, including a specification of covariates used for each model. After Bonferroni-adjustment, we found the following disease categories to be statistically significantly associated with documented physical child abuse: ischemic heart disease, RR 1.44 (CIc 1.13–1.84); peripheral artery occlusive disease, RR 1.39 (CIc 1.01–1.90); stroke, RR 1.19 (1.01–1.41); chronic pulmonary disease, RR 1.33 (CIc 1.18–1.51); ulcer/chronic gastritis, RR 1.23 (CIc 1.00–1.51); painful condition, 1.17 (CIc 1.00–1.37); epilepsy, RR 1.27 (CIc 1.03–1.56); unspecific symptoms within the last 2 years and ever, RR 1.31 (CIc 1.14–1.50) and RR 1.33 (CIc 1.17–1.52), respectively; and other diagnoses, RR 1.23 (CIc 1.09–1.39). Diabetes mellitus and chronic liver disease showed increased risks, but these were not statistically significant after Bonferroni correction.

**Table 5:** Model results for all disease categories (mark significant results with bold)

Cluster	Disease category	Relative risk, 95% and Bonferroni-corrected confidence intervals	
		Full models^a^	Parsimonious models^a^
Circulatory system	Hypertension	1.06 (0.95–1.17) (0.89–1.25)	1.06 (0.96–1.18) (0.90–1.25)
	Dyslipidemia	1.02 (0.91–1.15) (0.85–1.23)	1.02 (0.91–1.14) (0.85–1.22)
	**Ischemic heart disease**	**1.44 (1.24–1.68) (1.13–1.84)**	**1.44 (1.25**–**1.65) (1.15**–**1.80)**
	Atrial fibrillation	1.12 (0.93–1.34) (0.83–1.51)	1.14 (0.95–1.37) (0.84–1.54)
	Heart failure	0.83 (0.63–1.08) (0.53–1.28)	0.90 (0.70–1.15) (0.60–1.34)
	**Peripheral artery occlusive disease**	**1.39 (1.14–1.69) (1.01–1.90)**	**1.41 (1.15**–**1.72) (1.02**–**1.95)**
	**Stroke**	**1.19 (1.07–1.32) (1.01–1.41)**	**1.21 (1.10**–**1.35) (1.03**–**1.43)**
Endocrine system	Diabetes mellitus	1.11 (1.02–1.21) (0.97–1.27)	1.13 (1.04–1.22) (0.99–1.29)
	Thyroid disorder	1.01 (0.92–1.11) (0.87–1.17)	1.01 (0.93–1.11) (0.88–1.17)
	Gout	-	1.52 (1.12–2.07) (0.93–2.51)
Pulmonary system and allergy	**Chronic pulmonary disease**	**1.33 (1.23–1.44) (1.18–1.51)**	**1.35 (1.25**–**1.46) (1.20**–**1.53)**
	Allergy	0.95 (0.88–1.04) (0.83–1.09)	0.95 (0.88–1.04) (0.83–1.09)
Gastrointestinal system	**Ulcer/chronic gastritis**	**1.23 (1.08–1.40) (1.00–1.51)**	**1.31 (1.18**–**1.44) (1.11**–**1.53)**
	Chronic liver disease	-	1.55 (1.16–2.08) (0.97–2.49)
	Inflammatory bowel disease	1.05 (0.95–1.15) (0.89–1.22)	1.03 (0.94–1.13) (0.88–1.20)
	Diverticular disease of intestine	1.12 (0.94–1.32) (0.84–1.47)	1.12 (0.96–1.32) (0.87–1.46)
Urogenital system	Chronic kidney disease	0.90 (0.71–1.14) (0.61–1.32)	0.96 (0.78–1.18) (0.69–1.34)
	Prostate disorders	0.91 (0.66–1.25) (0.55–1.52)	0.91 (0.66–1.27) (0.54–1.55)
Musculoskeletal system	Connective tissue disorders	1.06 (0.95–1.19) (0.89–1.28)	1.07 (0.95–1.20) (0.88–1.29)
	Osteoporosis	0.89 (0.64–1.23) (0.52–1.51)	0.89 (0.65–1.22) (0.53–1.49)
	**Painful condition**	**1.17 (1.06–1.29) (1.00–1.37)**	**1.19 (1.07**–**1.31) (1.01**–**1.39)**
Hematological system	Anemias	1.09 (0.95–1.25) (0.88–1.35)	1.11 (0.97–1.27) (0.89–1.38)
Cancers	Cancer	0.98 (0.90–1.08) (0.85–1.14)	0.98 (0.89–1.07) (0.84–1.13)
Neurological system	Vision problem	1.16 (1.03–1.30) (0.96–1.39)	1.16 (1.04–1.30) (0.97–1.39)
	Hearing problem^b^	1.05 (0.96–1.15) (0.90–1.22)	1.05 (0.96–1.15) (0.90–1.22)
	Migraine	1.02 (0.92–1.14) (0.86–1.22)	1.01 (0.92–1.12) (0.86–1.19)
	**Epilepsy**	**1.27 (1.12–1.44) (1.03–1.56)**	**1.28 (1.13**–**1.46) (1.05**–**1.57)**
	Parkinson’s disease^c^	-	1.61 (0.60–4.30) (0.33–7.90)
	Multiple sclerosis	0.98 (0.80–1.21) (0.71–1.37)	0.96 (0.79–1.18) (0.70–1.33)
	Neuropathies	1.05 (0.94–1.18) (0.88–1.27)	1.05 (0.94–1.17) (0.88–1.25)
Unspecific symptoms	**Unspecific symptoms, two years back**	**1.31 (1.20**–**1.42) (1.14**–**1.50)**	**1.32 (1.21**–**1.44) (1.15**–**1.51)**
	**Unspecific symptoms, ever**	**1.33 (1.23**–**1.44) (1.17**–**1.52)**	**1.34 (1.24**–**1.45) (1.17**–**1.53)**
Other diagnoses	**Other**	**1.23 (1.14**–**1.32) (1.09**–**1.39)**	**1.23 (1.14**–**1.33) (1.09**–**1.39)**

Bold indicates 95% confidence interval not including 1, with and without Bonferroni adjustment

^a^A full model was adjusted for family income, neighborhood resources, immigration background, status as refugee, calendar time group, family highest education, parental psychiatric disease, inter-parental violence, parental substance abuse, and parental maltreatment in childhood; a parsimonious model was adjusted for family income, neighborhood resources, calendar time group, family highest education, parental psychiatric disease, inter-parental violence, parental substance abuse, and parental maltreatment in childhood. A dash (-) marks a model that did not converge

^b^A time trend of this category showed an unexpected and unexplained sudden increase in number of diagnoses on the 1st of January 2012. We have not found a practical explanation for this. Sensitivity analyses (not shown) on time before and after showed a significant confidence interval both before and after the shift. However, the confidence intervals were close to 1 and might have been spurious findings. Here the result shown is for the full period

^c^Because of a small number of exposed, Parkinson’s disease was only adjusted for income, calendar time group, and family highest education

## Discussion

In models adjusted for known risk factors for physical child abuse, the majority of disease categories explored were found to have no statistically significant association to documented physical child abuse. Ischemic heart disease, peripheral artery occlusive disease, stroke, chronic pulmonary disease, ulcer/chronic gastritis, painful condition, epilepsy, unspecific symptoms, and the broad category of “other diagnoses” showed varying degrees of associations. Diabetes mellitus and chronic liver disease showed increased risks, but results were not statistically significant after Bonferroni correction. Some findings of no association were expected based on our study on parental physical disease severity using the same dataset [12], published concurrently in this journal.

Motivating this study, we assumed that parental physical health influence family stress levels. As referenced earlier, this is indicated among parental unspecific symptoms [6] and functional somatic syndromes [16–18]. For many categories of disease, the specific impact on family stress levels is unknown, although there are studies on family functioning and its components. A previous study found no association between hypertension and family cohesion [54]. In diabetes, the association is influenced by treatment outcome. Among adults with non-insulin–dependent diabetes mellitus, good glycemic control was associated with lower family cohesion compared to those with lower quality glycemic regulation, while this relationship was reversed among adults with insulin-dependent diabetes mellitus [55]. Among chronic obstructive pulmonary disease patients, family functioning was found to be comparatively better among patients treated with oxygen in their home than hospitalized patients [56]. Lower family cohesion was found among individuals diagnosed with epilepsy compared to controls [57]. In a study of cancer, no differences were found in family functioning between patients and controls [58]. Headache symptom severity has been shown to adversely influence family cohesion [59]. Only cautious interpretations should be derived from single studies in each category. Nonetheless, it seems likely that disease categories’ influence on family functioning is not uniform, sometimes counterintuitive, and mediated by other factors, including treatment. Through this lens, the differing associations across categories in our results could be an expression of differences in how disease categories influence family stress.

The disease categories found to be associated with documented physical child abuse are all either chronic conditions or with considerable chronic subgroups. Consequently, part of the associations found could be an expression of the underlying chronicity of these categories. Nonetheless, a number of other disease categories representing chronic conditions show no association, possibly underlining the varying influences by different categories discussed above.

Residual confounding may also have influenced our findings. For example, adjusting for only two levels of education could result in strata with quite heterogeneous populations, and there may therefore be residual bias in the models. In addition, the model does not adjust for all possible risk factors. For example, the association with ischemic heart disease, peripheral artery occlusive disease, stroke, and chronic pulmonary disease may be affected by smoking. Smoking is a risk factor for all these conditions and has been associated with both childhood maltreatment among smokers [60] and as a risk factor for physical child abuse [29]. This is supported by the association before Bonferroni correction of diabetes mellitus, which is also associated with smoking. Thus, diseases resulting from smoking could be proxies for residual confounding from parental childhood maltreatment. Similarly, stomach ulcer/chronic gastritis is associated with substance abuse, which is also both a risk factor for [8] and a possible result of child maltreatment [61]. This is supported by the association of chronic liver disease without Bonferroni correction, also associated with substance abuse.

Epilepsy has been associated with depression and difficulties in emotional regulation [62], which may contribute to its influence on family cohesion. Affective disorders in parents have been linked to lethal physical child abuse [7, 8]. Another possible link could be that the risk of epilepsy is increased after trauma [63]. Thus, for a subgroup of parents with posttraumatic epilepsy, parental experiences of childhood maltreatment, which is a risk factor for maltreatment of offspring, could be proxied by adult epilepsy.

Chronic pain and childhood maltreatment have been previously studied, but Marin et al. in 2021 in a systematic review considered current evidence inconclusive [64]. The painful condition category in our study is based on the use of prescription analgesics at least four times within the last year. This category stands out by being defined by analgesics alone and thus possibly overlaps with other disease categories. Importantly, some diagnoses in the category unspecific symptoms describe manifestations of pain, and there might be a substantial overlap between the painful condition and unspecific symptoms categories.

Among unspecific symptoms, functional somatic syndromes have been shown to increase with childhood maltreatment [65]. This has also been shown for unspecific somatic complaints and childhood physical abuse, again invoking the possibility of residual confounding [66].

Unspecific symptoms stand out if their prevalence is taken into account. A better understanding of the individuals in the unspecific symptoms category, which encompassed 72% of the entire dataset, could potentially provide targets for preventive measures both to target populations and to inspire components for blended interventions. The current results do not warrant targeted interventions in their own right, but may inspire to future interventions such as integrating parental physical symptom checklists into existing home visiting programs or parenting programs or introducing tools to support parents in coping with their symptoms. However, further research is needed to study the associations found here and further specify their causal and/or predictive nature.

The association with the category “Other diagnoses,” diagnoses that do not fit with the remaining categories, is intriguing, but difficult to interpret due to their heterogeneous nature. Splitting this category into subcategories such as rare endocrine disorders, hereditary disorders, or similar may provide further insights.

Each disease category was not adjusted for other categories. This was inspired by our findings [12] as discussed above. Adjusting for other disease categories might consequently introduce more complexity to our models without a clear theoretical justification. Nonetheless, it is possible that some combinations, perhaps combinations of the categories found to be associated with documented physical child abuse, could have an additive or even multiplicative effect on the risk of abuse. This remains to be studied. Also, some diseases have common causes and may also be in each others’ causal pathways, for example, diabetes and heart disease [67]. This could explain the relatedness in our study of, for example, ischemic heart disease and stroke, and assumptions on the relations between categories should be remembered when designing future studies to elaborate on our results.

### Strengths and weaknesses

To the best of our knowledge, this is the first study to describe a diverse set of disease categories in parents and their link to child risk of documented physical child abuse. The longitudinal nature of the data and size of the sample enable discovery of relevant candidates for further scrutiny in predictive and causal models. The categories applied have been utilized previously, and additional categorization was done based on clinical insight. The models were adjusted for a number of known risk factors, showing the relevance of the associations even after taking current knowledge into account. There are also some potential weaknesses. The categories used were not validated, although a number of diagnoses in the registries used have been validated [48]. Although the assignment of ICD 10 codes in the Danish healthcare system is done by trained health professionals, the rate of irregularities for many diagnoses is not known. Such misclassification would be expected to be independent of the outcome and hence would bias toward no association. As mentioned above, the model may contain residual bias, from unobserved or insufficiently observed variables. This is because it is based on registry data; for example, substance abuse is based on hospital diagnoses only. Thus, as argued above, the associations seen could be hypothesized to be based on residual confounding. On the contrary, this is useful in its own right for predictions. If, for example, stomach ulcer/chronic gastritis captures a part of substance abuse that is not otherwise visible through either registries or clinical observation, this category is useful for predictive modeling. Additional categorizations of diagnoses were only done by the first author. Although the categorizations were checked rigorously and backed up by clinical experience, a lack of validation might lead to unexpected associations. As noted for Table 5, the category hearing problems has an unexplained behavior in the time trend of number of diagnoses. Consequently, results from this category should be interpreted with special caution. Number of missing entries at entry in source population on neighborhood resources and reconstituted family were 20% and 23%, respectively. All cohorts were drawn using only complete cases, and there is a risk of bias if the risk of missing data is associated with differences in the underlying population, possibly affecting generalizability of the results. We used data sets drawn from all children living in Denmark up until 2018 in this study, and in some categories, we used all available children exposed to a parental disease category (for details, see Table 4). Nonetheless, a population correction factor was not used to correct the confidence intervals presented. This was because we regarded our population as a sub-population of all children living in settings with universal healthcare and because no analyses used data on all available non-exposed children. Also, some disease categories may represent a subset of families with parents in these categories, both because a maximum of 100,000 exposed families were allowed in each analysis and because some disease categories, for example, hypertension, may be underdiagnosed in patient registries not including the primary sector. Consequently, our analyses never represented the full population of interest. If the population of interest was limited to Danish children only and the variation in remaining non-exposed children assumed to be negligible, categories expected to be fully diagnosed within the registries available to us could be presented with more precise confidence intervals. However, as we find the estimated strength of associations presented as insignificant in our analyses to be moderate or less (see relative risks in Table 5), the usefulness of such categories for preventive and predictive purposes would be expected to be limited. Finally, as mentioned above, this study took place in a high-income country [68] with universal healthcare coverage. Results are likely to generalize to similar populations but would require replication elsewhere, in particular in low-income countries and healthcare systems that are organized differently. Additionally, most self-reported cases of maltreatment are not known to the sources used for the outcome [69]. Consequently, results may not generalize to the cases of physical child abuse unknown to healthcare or law enforcement.

## Conclusion

A number of diverse disease categories were exploratively tested for an association with documented physical child abuse. A notable candidate for future studies was unspecific symptoms, presenting both a significant prevalence and an association with the outcome. Further candidates were ischemic heart disease, peripheral artery occlusive disease, stroke, chronic pulmonary disease, stomach ulcer/chronic gastritis, painful condition, and epilepsy. Further research into these is warranted and may inspire additions to preventive interventions.

### Supplementary Information

Below is the link to the electronic supplementary material.

Supplementary file1 (XLSX 230 KB)

## Data Availability

Access to data was granted by Statistics Denmark, and this study was exempt from ethics approval according to Danish law, as it only used administrative data. Data could not be made available due to legal requirements by Statistics Denmark. All code used, and additional resources, is available at https://osf.io/fh2sr.

## Abbreviations

ATC: Anatomical Therapeutic Chemical
CI: 95% Confidence interval
CIc: Bonferroni-corrected 95% confidence interval
ICD 8: International Classification of Diseases, 8th edition
ICD 10: International Classification of Diseases, 10th edition
ISCED: International Standard Classification of Education
N: Number of units
RR: Relative risk
SD: Standard deviation
